# Explainable AI and Multi-Modal Causability in Medicine

**DOI:** 10.1515/icom-2020-0024

**Published:** 2021-01-26

**Authors:** Andreas Holzinger

**Affiliations:** Human-Centered AI Lab, Institute for Medical Informatics & Statistics, 31475 Medical University Graz, Graz, Austria; xAI Lab, 530945 Alberta Machine Intelligence Institute, Edmonton, Canada

**Keywords:** explainable AI, Human-Centered AI, Human-AI interfaces

## Abstract

Progress in statistical machine learning made AI in medicine successful, in certain classification tasks even beyond human level performance. Nevertheless, correlation is not causation and successful models are often complex “black-boxes”, which make it hard to understand *why* a result has been achieved. The explainable AI (xAI) community develops methods, e. g. to highlight which input parameters are relevant for a result; however, in the medical domain there is a need for causability: In the same way that usability encompasses measurements for the quality of use, causability encompasses measurements for the quality of explanations produced by xAI. The key for future human-AI interfaces is to map explainability with causability and to allow a domain expert to ask questions to understand why an AI came up with a result, and also to ask “what-if” questions (counterfactuals) to gain insight into the underlying *independent* explanatory factors of a result. A multi-modal causability is important in the medical domain because often different modalities contribute to a result.

## 1 Introduction: Deep Learning Success Examples in Medical AI

To reach human-level AI is the quest of AI researchers since the emergence of this field [1]. Research in the last decades has proved it to be very difficult and the progress has been slow, despite great success in statistical machine learning theory [2] and statistical learning practice [3]. Recently, there have been many practical successes of deep neuronal network learning [4] due to the availability of large amounts of training data sets and computational power. In the health domain there are many different areas, where AI can help, e. g. in diagnostics and decision making, drug discovery, therapy planning, patient monitoring, risk management, areas dealing with “big data” such as the analysis of *omics data, including genomics, proteomics, metabolomics, and many others [5]. One particular relevant field is medical image analysis, including AI-applications in pathology [6], radiology [7], dermatology [8], ophthalmology [9], oncology [10], and many other medical application fields.

Let us look at a meanwhile classic work, presented in 2017 by the group of Sebastian Thrun from Stanford, which was sold under “Beyond human-level performance” [11] and was popularized in the news in Europe as “AI is better than doctors”. What did they do? They classified skin lesions using a single convolutional neural network (CNN), trained it end-to-end from the derma images directly, and used only pixels and disease labels as inputs. For pretraining they used 1.3 million images from the 2014 ImageNet challenge. Then, they used 130 thousand clinical images consisting of approximately 2000 different diseases, reaching 92 % average classification performance, on par with human dermatologists or even better. If we consider that the algorithm does not get tired this is really an amazing result, considered by medical doctors as a very good performance. However good these results may be, pressing questions are raised: *“Why can AI solve some tasks better than humans?”*, *“Why does the AI achieve such results?”*, *“Which underlying factors are contributing to the result?”*, or *“What if I change, replace, disturb, remove input data?”*, or more technically: *“What if the input data changes counterfactually …?”* This needs to consider and examine desired properties of methods, including *fidelity* [12], [13], *interpretability* [14], *parsimony* [15], and *generalizability* [16].

A very recent work from the Princess Margaret Cancer Center in Toronto in the field of histopathology [17] goes one step in this direction: They also applied a CNN to a repository of 840 thousand histopathological image tiles and learned representations into a 512-dimensional feature vector. The novelty here is that they showed that machine-generated features correlate with certain morphological constructs and ontological relationships generated by humans. Why is this important for us? Because highlighting such overlaps between human thinking and machine “thinking” can contribute to what are currently top issues in the machine learning community: i) to eliminate bias and to improve algorithms robustness, and ii) to make the results retraceable, hence explainable in order to meet the quest of accountability of medical AI.

Despite all these successes, one of the most pressing problems is in robustness, i. e. in overcoming the “brittleness” of current AI systems, because true human-level AI requires computational approaches that are able to deal with “common sense” situations [18] and to “think” and “act” like humans. Many advances have resulted from using deep neural networks trained end-to-end in such tasks. Despite their biological inspiration and the impressive results mentioned before, these systems differ from human intelligence enormously. Besides lacking robustness and generalization, current approaches are unable to build causal models in order to support deep understanding [19]. Consequently, to make such approaches even more successful we need further work to make them *robust* [20], [21], *understandable* [22], and *interpretable* for a human expert [14]. The aim is to take advantage of the respective benefits of both statistical machine learning methods and model-based approaches, or more precisely: The aim is to integrate existing a-priori knowledge and human experience into statistical learning methods, thereby combining them synergistically in a hybrid approach to exploit the full benefits of data-driven methods without ignoring already acquired knowledge an human expertise. Here, a human-in-the-loop can be (sometimes, of course not always) helpful as we will discuss in section 3. Before that, we briefly discuss some basics of explainability and causability.

## 2 Explainability and Causability

The field of explainable AI is meanwhile very popular [23], [24], [25], [26], and the explainable AI (xAI) community is very active in developing various methods to help making such “black box” approaches, as outlined in the introduction, retraceable, understandable, and human interpretable.

It is important to note that results are interpretable when they classify objects on the basis of features that *a human can understand* [27]. Current approaches to explaining the decisions of deep learning for medical tasks have focused on visualising the elements that have contributed to each decision, which can be done e. g. via interactive heatmaps [28], [29]. Such “mechanical explanations” to highlight which input is relevant to an obtained output can be reached by using various methods: The simplest method works with gradients as multi-variable generalization of the derivative, where the neural network is seen as a function and the explanation relies on the function’s gradient, which is available from the backpropagation algorithm [30]. Another possibility is to use decomposition methods (luckily our world is compositional), e. g. pixel-wise relevance propagation [31], layer-wise relevance propagation [32], or deep Taylor decomposition [33], which also works on graph-based data [34]. Other methods include deconvolution by reversing the effects of convolution and bringing out from two functions a third function which is then the product of both, guided backpropagation, and the use of so-called concept activation vectors [35], [36], [37], [38].

All these methods are excellent pre-processing steps, however, in a way that a medical expert can understand the *causality* of a learned representation and use it for *medical decision support* the xAI methods need to be developed even further. Let us note that xAI (or “explainability”) deals with the implementation of methods to enable retraceability, transparency, and interpretability of so-called “black‐box” methodologies. The currently best performing methods, as we have seen in the best-practice examples in the introduction above, are of such kind. Unfortunately, it is not an option just to say *“stop explaining black-box machine learning models for high stakes decisions and use interpretable models instead”* as stated by Cynthia Rudin [14], because this would mean not to use the currently best performing methods.

However, in the biomedical domain there is a need to go beyond xAI. To reach a level of “explainable medicine” there is a crucial need for causability [39]. In the same way that usability encompasses measurements for the quality of use, causability encompasses measurements for the quality of explanations, e. g. the heatmaps produced by explainable AI methods. Causability can be seen as a property of “human intelligence”, whereas explainability can be seen as the property of a “artificial intelligence”.

The key to effective human-AI interaction and consequently the success of future human-AI interfaces lies in an efficient and consistent **mapping of explainability with causability** [40].

This “mapping” is about establishing connections and relationships between existing areas, so *not* about drawing a new map, but rather to identify similar areas in two completely different “maps”. Effective and efficient mapping is necessary, but obviously not sufficient for understanding an explanation: Whether an explanation has been understood depends on other factors, including prior knowledge and expectations on the human side. Obviously, the effectiveness of an “explanation interface” depends on whether (and to what extent) the result of an explanation produced by an explainable AI method was understood by the human expert.

As we can imagine this is not trivial, because future Human-AI interfaces should allow a constant feedback, whether and to what extent something has been understood, or not. In a human-to-human interaction, this feedback is very much provided by facial expressions. Consequently, concepts of “emotion” [41], [42], “emotion detection” [43] and “emotional interfaces” [44] will become an important part of future conversational interfaces for explainable AI [45] and dialog systems [46]. Such features will become important for these future “explanation interfaces” or however we will call them. One very important aspect is to include a key component that has been used as a standard communication tool between doctors for centuries: *language*, i. e. to produce descriptive sentences based on *domain ontologies* to clarify the decision of deep learning classifiers, hence to augment the results with short quantified sentences of natural language [47].

Summarizing, humans will continue to play a special role in the AI pipeline in the foreseeable future, complementing capabilities of AI due to their genuine human abilities. The backbone of this approach is interactive machine learning [48], [49], [50] which adds the component of human expertise to AI processes by enabling them to re-enact and retrace the results on demand, e. g. let them check it for plausibility.

## 3 Towards Future Human-AI Interfaces for Multi-Modal Causability

We can recapitulate that in the medical domain we need to include a human-in-the-loop for several reasons: to complement AI with human expertise and conceptual knowledge, to augment the human with AI, and also to keep the human in control for social, ethical and legal reasons [51]. For all these reasons there is a pressing need for the design, development, and evaluation of new effective and efficient Human-AI interfaces. This challenges the Human-computer interaction (HCI) community: Design guidelines for human-AI interaction are already under way [52]. Moreover, general principles and design guidelines for interactive techniques have been discussed in the HCI community for decades [53], which are now becoming important again. Lastly, the quest for effective Human-AI interfaces was boosted recently by the xAI program of DARPA, where they explicitly emphasized the importance of *interactive “explanation interfaces”* [54] and where they emphasized that understanding (sensemaking) must be facilitated by *interactive* guided explanations. This is motivated by the fact that for a biomedical expert using AI, it is very important to be able to investigate the **independent** underlying factors which influenced the machine aided decision-making process, taking into account that we cannot always disentangle dependent factors. That said, decision paths defined by biomedical experts will capture *only a subset of the features available* to train machine learning models in medical AI. From this reduced feature set (multi-*omics and clinical parameters), it can be beneficial to build reference classification models based on decision trees which may reflect the biomedical decision process. Such decision trees can then act as a reference model, but most importantly, as a benchmark for the *reliability* of “black-box” AI models. We need to carefully study the accuracy of such reference models and to investigate their generalizability regarding heterogeneous patient profiles. In this context, disease subtypes can be derived. For this purpose, the development of new and the application of existing multi-view clustering algorithms [55] can be very helpful.

A very useful approach to combine various data in order to create comprehensive views of diseases or biological processes is *Similarity Network Fusion* (SNF) developed by Wang et al. [56]. This method solves the integration problem by constructing networks of samples for each available data type and fusing these into one single network that represents the underlying data. The increasing complexity of the biomedical domain and the introduction of new technologies enable investigations in arbitrarily high dimensional spaces, practically having millions of different properties (including genomics and proteomics, but also images, patient history, etc.). No single data type can, however, capture the complexity of all these factors which are relevant to understand a phenomenon, i. e. a disease. This calls for integrative methods that combine data from multiple technologies and provide a comprehensive and relevant system view [57], [5], [58].

An ideal method must be able to “answer” a biological or medical question, i. e. to identify important features and predict outcomes, by harnessing heterogeneous data across several dimensions of biological variation. Very useful in this context is Neighbourhood based Multi-*omics clustering (NEMO) [59]. NEMO can be applied to partial datasets without performing data imputation and works in three phases: First, an inter-patient similarity matrix is built for each *omics data; then the matrices of different *omics data are integrated into one single matrix; finally this network is clustered. A very recent approach is *Pathway Graph Kernel based Multi-Omics Approach for Patient Clustering* (*PAMOGK*) [60], that integrates multi-*omics patient data with existing biological knowledge on pathways. A graph kernel evaluates patient similarities based on a single molecular alteration type in the context of such a pathway, and to support multiple views, a multi-view kernel clustering is used. A measurement for the predictive power is the *Area under the Curve* (AUC). In the context of explainability/causability, however, only parts of the AUC are informative. This is mostly due to the fact that we are often confronted with imbalanced data in the biomedical domain [61]. Known alternatives such as the partial AUC cannot be fully interpreted, because they ignore some information about actual negatives. However, the recently developed concordant (partial-) pAUC is more useful [62] and may help to understand and interpret parts of the AUC.

Although the above mentioned models perform well, we are far from being able to use them within daily biomedical practice as long as *the underlying decision paths are not made visible*, and most importantly, *understandable and interpretable* for the end-user, because we still are confronted with the “black-box problem” [63]. Here we should note that the decision-making process can be seen as a sequence of steps in which the biomedical expert selects a path through a network of *plausible* events and actions. This goes back to the seminal work of Shortliffe et al. [64]: Nodes in this tree-shaped network are of two kinds: “decision nodes”, where the expert can select from a set of actions, and “chance nodes”, where the outcome cannot be directly controlled by the expert, but is a probabilistic response of the patient to some action taken. For example, a physician may choose to perform a certain test (decision node) but the occurrence or non-occurrence of complications may be largely a matter of statistical likelihood (chance node). By analyzing a difficult decision process before taking any action, it may be possible to delineate in advance all pertinent decision nodes and chance nodes along with all plausible outcomes, plus the paths by which these outcomes might be reached. To address this shortcoming, one possibility is to relate the multi-modal models, which are built on stochastic procedures only, to a biomedical expert’s reference model. This requires investigating whether and to what extend the corresponding decision paths are reflected and/or covered. This can be done via *“what-if”* (*counterfactuals*) *requests* to the system, but also with additional *state-of-the-art* approaches that are widely used by the xAI community to date, for example, popular model-agnostic approaches such as DALEX [65], LIME [66], or, more recently, optiLIME [67]. All these approaches can be used for both global explainability (for model understanding) and for local explainability (for prediction understanding). Every explainer creates a numerical summary and a visual summary and allows for comparison of multiple models. To enhance the understandability for the domain expert, this can be augmented via *short quantified sentences on natural language* [47]. A big advantage of the counterfactual generation is that it can be considered as a post-hoc procedure which can act independent from any classifier [68]. The resulting counterfactuals can be modelled as a graph, where features are defined as nodes and the edges as combination of such features, which we call *“counterfactual paths”*. Initially, such a counterfactual graph may be generated in a purely data-driven manner. The distance between the counterfactuals (weighted edges) can be defined as in [69]. In an ideal setting, the automatic generation of the counterfactual paths is fully reflected by the leaf nodes of the medical decision trees [70]. To facilitate the human *interaction* with the multi-modal machine learning model opens new ways of interactive Human-AI interface, supporting both explainability and causability. Here, the guiding idea is that the biomedical experts are empowered to ask questions (“why are the cells smaller and closer together”) and also counterfactual “what-if” questions (“what if the cells are slightly bigger”). Here a chance is to derive *simple-to-understand* decision trees derived from the graph, which itself can be derived by a decision forest classifier comprising multiple trees derived from the counterfactual classes. Recent work has shown how to efficiently reduce such a decision forest to a single decision tree [71], [72] from which counterfactuals can be easily observed based on the leaf nodes. Here. the human in the loop will have the opportunity to study this consensus decision tree. and the domain expert will be able to adopt the modifications to the counterfactual graph accordingly (feedback-loop). It is necessary to visualize relevant input features as well as the underlying explanatory factors, the “decision network”. This is of course a non-trivial task because such a visualization has to be optimized to a) the human visual perception capability, b) the prior knowledge of the human, and c) the context of the workflow. This calls for *flexible* interfaces, taking into consideration existing methods, algorithms and tools [73], [74], from a) post-hoc interpretable models and systems, which aim to provide local explanations for a specific decision and making it reproducible on demand (instead of explaining the whole model behaviour), over b) ante-hoc models, which are interpretable by design which includes so called glass-box approaches [50]. Of course, a lot of further research in the real-world is needed regarding the technical parameters’ robustness and explainability.

## 4 Conclusion

Thanks to the great progress in statistical learning, we are experiencing an AI renaissance. Available and practical useable deep learning approaches achieve a performance that is beyond human level performance – even in the medical domain. This is a great success and there is no question that AI will become very important for medicine. Especially, when considering what humans are *not* able to do – but AI can. Nevertheless: correlation is not causation and contemporary AI models have become so complex that they are considered as “black-box” approaches. This makes it hard for domain experts to understand why a certain result has been achieved. The xAI community has developed a variety of methods for making such approaches transparent. This constitutes a promising first step, but while xAI deals with the implementation of transparency and traceability in statistical black‐box machine learning methods in the medical domain, there is a pressing need to go beyond xAI: to reach a level of explainable medicine we need causability, which encompasses measurements for the quality of explanations produced by xAI methods (e. g. heatmaps). Here, very important is the human in the loop, because (sometimes) a human expert is necessary to add contextual understanding and experience. This, in turn, requires new ***interactive***human-AI interfaces, especially in the medical domain, in which many different modalities contribute to a result. To support future “explainable medicine”, we therefore need multi-modal causability. That said, we need interactive Human-AI interfaces which enable a domain expert to ask questions to understand why a machine came up with a result, and to ask “what-if” questions (counterfactuals) to gain insight into the underlying independent explanatory factors of a result. Overall, intensive research and development on an international level will thus be necessary in to make AI even more successful and to use medical AI effectively for the benefit of human health.
